# Contemporary Management of the Aortic Arch: A Narrative Review

**DOI:** 10.3390/jcm15114137

**Published:** 2026-05-27

**Authors:** Nafiye Busra Celik, Danial Ahmad, Asad S. Fatimi, Sriharsha Talapaneni, Mahid Qureshi, Irbaz Hameed, Prashanth Vallabhajosyula

**Affiliations:** Division of Cardiac Surgery, Department of Surgery, Yale School of Medicine, New Haven, CT 06519, USA; nafiyebusra.celik@yale.edu (N.B.C.); danial.ahmad@yale.edu (D.A.); asad.fatimi@scholar.aku.edu (A.S.F.); sriharsha.talapaneni@yale.edu (S.T.); mahid.qureshi@yale.edu (M.Q.); irbaz.hameed@yale.edu (I.H.)

**Keywords:** aortic arch, Ishimaru zones, Frozen Elephant Trunk (FET), TEVAR, branched/fenestrated endovascular repair, antegrade cerebral perfusion, open arch repair

## Abstract

The aortic arch remains one of the most complex segments of the thoracic aorta to treat, demanding strategies that safeguard cerebral and spinal perfusion while achieving durable proximal and distal repair. Contemporary management strategies include open hemi/total arch replacement, hybrid approaches such as frozen elephant trunk (FET) or debranching with thoracic endovascular aortic repair (TEVAR), and fully endovascular repair using branched or fenestrated devices. Updated guidelines (American College of Cardiology/American Heart Association [ACC/AHA] 2022; European Society of Cardiology [ESC] 2024) emphasize multidisciplinary, patient-specific decision-making grounded in standardized imaging, genetics, and lifelong surveillance. Procedurally, selective antegrade cerebral perfusion with moderate-to-low hypothermia has replaced routine deep hypothermic circulatory arrest for most open arch operations. Zone-based planning using Ishimaru’s map, complemented by the Modified Arch Landing Areas Nomenclature (MALAN), improves feasibility assessment and risk stratification, while entry-focused schemas like TEM (Type, Entry, Malperfusion) further refine management. Emerging data indicate that open repair remains the durability benchmark in younger populations and those with connective tissue disease, and FET enables single-stage treatment capability with acceptable early outcomes but requires vigilant neurologic protection and reintervention surveillance. An integrated, zone-driven approach guided by center expertise optimizes patient selection for open, hybrid, or endovascular options to maximize safety and durability.

## 1. Introduction

The aortic arch remains one of the most technically challenging sections of the thoracic aorta to treat given its critical role in maintaining uninterrupted cerebral and spinal cord perfusion and its involvement in a wide spectrum of pathologies, including aneurysm, dissection, intramural hematoma, and penetrating aortic ulcer. Surgical and endovascular interventions in this region must therefore balance the competing priorities of durable aortic repair and protection of vital organ perfusion, making treatment decisions uniquely challenging.

Over the last decade, management has undergone a significant paradigm shift. Traditional open surgical approaches, including hemi- or total arch replacement, remain the reference standard for durability, particularly in younger patients and those with heritable aortopathies. However, the therapeutic landscape has expanded to include hybrid strategies such as the frozen elephant trunk (FET) technique and, increasingly, fully endovascular alternatives with branched and fenestrated devices. These developments have broadened the spectrum of treatable patients, particularly those previously considered high risk for conventional surgery, and have introduced new considerations in procedural planning, risk stratification, and long-term surveillance.

Contemporary management is increasingly guided by a multidisciplinary, patient-centered framework that integrates clinical presentation, aortic pathology, anatomic complexity, and institutional expertise. Recent guidelines, including the 2022 American College of Cardiology/American Heart Association (ACC/AHA) and 2024 European Society of Cardiology (ESC) recommendations, emphasize standardized imaging, genetic evaluation, and lifelong follow-up, reflecting a shift toward more structured and individualized care pathways [1,2]. In parallel, advances in operative technique, especially the adoption of selective antegrade cerebral perfusion with moderate-to-low hypothermia, have improved neurologic outcomes and refined the safety profile of open arch surgery [3].

At the same time, rapid progress in endovascular technology has redefined the boundaries of minimally invasive arch repair. The development of branched and fenestrated endografts, including off-the-shelf platforms such as the GORE TAG Thoracic Branch Endoprosthesis (TBE) and evolving systems like Nexus and RelayBranch, has enabled treatment across multiple arch zones with preservation of supra-aortic branch perfusion [4,5,6]. These innovations, together with advances in hybrid techniques, have created a complex and evolving treatment landscape in which the optimal strategy is increasingly individualized.

The aim of this review is to provide a recent, clinically focused overview of aortic arch management, encompassing open surgical, hybrid, and fully endovascular approaches. In addition, we highlight key considerations that guide decision-making in modern practice, including patient selection, anatomic assessment, procedural strategy, and neurologic protection.

## 2. Literature Search

This article is a narrative, expert-driven review intended to provide a modern overview of aortic arch management. Relevant literature was identified through targeted searches of PubMed/MEDLINE and review of reference lists of key articles in November 2025. Search terms included combinations of “aortic arch,” “open arch repair,” “frozen elephant trunk,” “TEVAR,” “branched endovascular repair,” and “cerebral perfusion.” Emphasis was placed on contemporary guidelines, randomized trials, high-quality observational studies, and major registry data. Given the narrative design, a formal systematic search strategy with predefined inclusion and exclusion criteria was not performed, and the findings should be interpreted in the context of potential selection bias.

## 3. Anatomy and Classification

**Figure 1:**
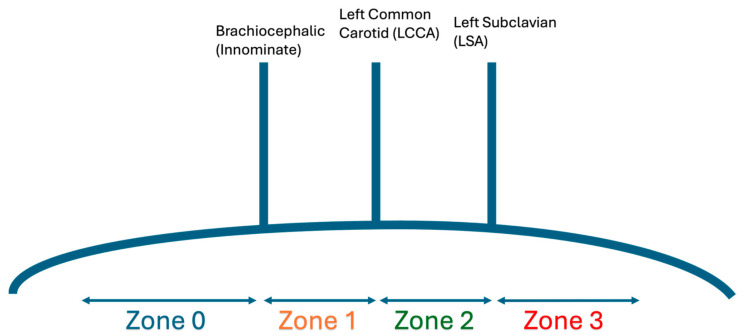
Ishimaru Aortic Arch Landing Zones (0–3). **Zone 0**: proximal to innominate origin; **Zone 1**: between innominate and LCCA; **Zone 2**: between LCCA and LSA; **Zone 3**: distal to LSA into proximal descending thoracic aorta.

Comparing techniques across studies and devices requires a precise, common nomenclature. Therefore, Ishimaru’s aortic arch map remains the reference framework for endovascular and hybrid planning by dividing the arch and proximal descending thoracic aorta into landing zones 0–3 according to the origins of the supra-aortic branches (Figure 1). This technique enables a concise description of proximal and distal zones for TEVAR and branched/fenestrated constructs [7].

The Modified Arch Landing Areas Nomenclature (MALAN) complements Ishimaru by associating the landing zone with arch type I-III because proximal landing feasibility also depends on arch geometry such as angulation, curvature, and stent-graft drag forces. Clinical studies suggest that MALAN can identify “hostile” arch segments with higher displacement forces and less favorable results following TEVAR. Accordingly, its use in preoperative planning, particularly in the context of debranching with TEVAR, frozen elephant trunk (FET), or branched arch devices, facilitates more accurate risk stratification [8,9].

In parallel, there has been a shift toward entry-focused classification systems for aortic dissection. The TEM (Type, Entry, Malperfusion) classification emphasizes the location of the primary entry tear and the presence of malperfusion, providing additional prognostic and therapeutic insight beyond traditional extent-based (Stanford/DeBakey) systems. This approach is particularly relevant in non-A non-B aortic dissections, where the primary entry tear originates within the aortic arch and does not conform to classical ascending or descending patterns. These lesions often demonstrate distinct clinical behavior, including higher risks of malperfusion and aortic progression, and may necessitate arch-directed strategies such as total arch replacement, FET, or branched endovascular repair. Recent guidelines and reporting standards from the 2024 European Association for Cardio-Thoracic Surgery/Society of Thoracic Surgeons (EACTS/STS) “Aortic Organ” guideline and the Society for Vascular Surgery/Society of Thoracic Surgeons (SVS/STS) therefore emphasize accurate identification of the entry tear and integration of entry-based classification with zone-based planning to guide management and improve comparability across studies [10,11].

## 4. Advanced Preprocedural Planning

Preprocedural planning has become increasingly sophisticated in contemporary aortic arch intervention, particularly with the expansion of hybrid and fully endovascular techniques. High-resolution computed tomography with three-dimensional reconstruction remains the foundation for procedural planning, enabling detailed assessment of arch geometry, landing zones, branch vessel orientation, and access feasibility. Beyond conventional imaging, advanced tools such as computational modeling, patient-specific three-dimensional printing, and virtual or mixed reality platforms are emerging as adjuncts to improve procedural planning and simulation [12]. These technologies allow for enhanced spatial understanding of complex arch anatomy, facilitate device selection and sizing, and may enable rehearsal of challenging cases prior to intervention. Early experience with holographic and mixed reality systems in structural and endovascular interventions suggests potential benefits in procedural accuracy, team communication, and training [13]. As these tools continue to evolve, they are likely to play an increasingly important role in the planning and execution of complex aortic arch procedures [12,13].

## 5. Management Approaches

### 5.1. Open Arch Replacement

Open hemiarch or total arch replacement (OAR) remains the reference approach for durable treatment of aortic arch pathology despite the growth of endovascular approaches especially in younger patients, connective tissue disease, hostile arch anatomy, or when long proximal/distal reconstructions are required. Recent guidelines emphasize individualized, team-based selection, precise imaging, and lifelong surveillance [1,2].

In addition, Acute Type A aortic dissection remains the most common urgent indication for aortic arch intervention and introduces additional complexity in operative decision-making [14]. The choice between hemiarch and total arch replacement is influenced by factors such as the location of the primary intimal tear, extent of arch involvement, presence of distal malperfusion, and patient stability [15,16]. Hemiarch replacement remains appropriate in cases where the primary tear is confined to the ascending aorta with limited arch involvement, offering shorter operative times and reduced physiologic burden in unstable patients. In contrast, total arch replacement may be favored when the primary entry tear involves the arch or when there is extensive arch destruction, improving long-term aortic remodeling at the expense of increased operative complexity.

Furthermore, the need for hypothermic circulatory arrest (HCA) with its associated risks is a major challenge for the open repair of the aortic arch. Overall, the brain is prone to ischemia in the short term because of its high metabolic demand and need for aerobic energy production. Therefore, neurologic protection has shifted toward selective antegrade cerebral perfusion with moderate-to-low hypothermia to provide continuous flow to the brain rather than routine deep hypothermia. A multicenter randomized trial (GOT-ICE) showed noninferiority of moderate-to-low hypothermia to traditional deep hypothermia for clinical outcomes and postoperative cognitive change after arch surgery, as informed by the current perfusion targets [3,17].

In addition to advances in perfusion strategy, intraoperative cerebral monitoring has become an integral component of modern aortic arch surgery. Near-infrared spectroscopy (NIRS) is widely used for real-time assessment of regional cerebral oxygenation and can guide adjustments in perfusion strategy. Electroencephalography (EEG) provides information on cortical activity and adequacy of cerebral protection, particularly during circulatory arrest. Transcranial Doppler may also be utilized in specialized centers to assess cerebral blood flow dynamics and detect embolic signals. These modalities, used in conjunction with selective antegrade cerebral perfusion, contribute to improved neurologic outcomes by enabling timely intraoperative intervention.

**Figure 2:**
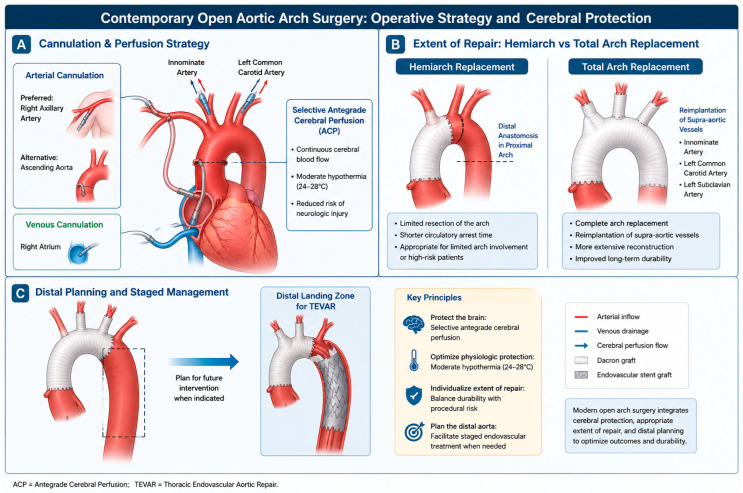
Operative strategies and cerebral protection in open aortic arch surgery.

A diagrammatic representation of operative strategies in open aortic arch surgery is presented in Figure 2.

### 5.2. Hybrid Arch: Frozen Elephant Trunk (FET) and Debranching + TEVAR

The FET technique combines open surgical arch replacement with a distal stented graft, enabling single-stage treatment of combined arch and proximal descending thoracic aortic pathology while creating a durable landing zone for potential downstream TEVAR. It is particularly useful in patients with extensive arch involvement, concomitant descending aortic disease, or acute Type A dissection with distal malperfusion, where it facilitates simultaneous proximal repair and stabilization of the downstream aorta [18,19]. This strategy may promote favorable aortic remodeling and reduce the need for early secondary interventions.

Contemporary series report 30-day mortality rates of approximately 5–12%, with improving neurologic outcomes in experienced centers [20,21]. However, these benefits must be balanced against procedure-specific risks, most notably spinal cord ischemia (SCI) and distal stent-induced new entry tears, particularly with more extensive distal aortic coverage.

Spinal cord protection is therefore a critical component of FET planning and perioperative management. Current strategies focus on maintaining adequate spinal cord perfusion pressure and minimizing ischemic burden. These include cerebrospinal fluid drainage to reduce intrathecal pressure, permissive hypertension in the perioperative period, avoidance of prolonged hypotension, and staged approaches in selected patients to allow for collateral network adaptation. Preservation of collateral circulation, particularly via the left subclavian artery, and careful attention to the extent of segmental artery coverage are also essential. In specialized centers, neuromonitoring with motor evoked potentials may facilitate early detection of spinal cord compromise.

Long-term and institutional data further underscore the importance of technical strategy. In a 15-year single-center experience, the reported paraplegia rate was 4.3%, with a notable reduction observed following adoption of a more proximal zone-2 arch strategy [22]. These findings highlight the influence of landing zone selection and distal coverage on neurologic outcomes, reinforcing the importance of individualized operative planning.

Alongside FET, hybrid arch repair using supra-aortic debranching combined with TEVAR (HAR) offers an alternative strategy, particularly in older or higher-risk patients in whom the physiologic burden of OAR is prohibitive. Comparative analyses suggest similar mid-term survival to total arch replacement, albeit with higher rates of late aortic reintervention. In contrast, open or FET-based approaches may be preferred in younger patients or those with dissection-predominant pathology, where long-term durability is a primary consideration [23,24].

### 5.3. Fully Endovascular Arch Repair (Branched/Fenestrated)

Endovascular strategies for the aortic arch have progressed from feasibility to targeted implementation at experienced centers, using custom fenestrated (fTEVAR) and branched (bTEVAR) platforms to maintain supra-aortic flow while securing proximal seal in zones 0–2. In a single-center series of 126 patients with a native proximal aortic landing (NPAL) treated with custom devices, technical success was 94.4%, with 30-day mortality of 11.9% and stroke rate of 13.5% (major 7.9%). Zone 0 landings independently increased stroke risk, and freedom from reintervention was 46.4% at 24 months, underscoring the neurologic risks of proximal arch sealing and the necessity of close surveillance [25].

Another comparative multicenter study showed that although both branched and fenestrated approaches achieve high technical success, safety signals may differ. Hauck et al.’s multicenter study (bTEVAR 20 patients versus fTEVAR 34 patients) showed perioperative mortality at 10% with bTEVAR versus 0% with fTEVAR. The major stroke rate was 10% with bTEVAR and 3% with fTEVAR, respectively in the same study. A total of 91% of fTEVAR cases would have been suitable for bTEVAR, whereas only 35% of bTEVAR cases were found suitable for fTEVAR, indicating that anatomical cross-applicability favored bTEVAR. This study also highlighted a trade-off between broader anatomic eligibility and potentially higher embolic risk with more proximal branched constructs [26].

The above-mentioned management approaches are succinctly summarized in Table 1.

## 6. Patient Selection

Decision-making in aortic arch management is best conceptualized as a structured, stepwise process integrating patient, disease, anatomic, and institutional factors. Patient-related considerations, including age, frailty, prior sternotomy, and the presence of heritable thoracic aortic disease, are central in determining procedural tolerance and long-term durability requirements. In general, younger patients and those with connective tissue disorders are preferentially considered for open surgical repair given its superior durability, whereas older or higher-risk patients may be better suited to hybrid or endovascular approaches.

Disease-related factors, including pathology type, including aneurysm, penetrating aortic ulcer (PAU), intramural hematoma (IMH), or acute or chronic dissection, and urgency of presentation, further refine strategy selection. Acute Type A aortic dissection often necessitates urgent open repair, with the extent of arch intervention guided by entry tear location and distal involvement, while degenerative aneurysmal disease or chronic dissections may allow for more elective, anatomy-driven planning.

Anatomic considerations are equally critical, including Ishimaru landing zones, arch geometry (as described by MALAN), proximal and distal seal length, and vascular access. These factors determine the feasibility of endovascular or hybrid strategies and influence the risk of complications such as stroke or spinal cord ischemia.

Finally, institutional expertise and device availability play a defining role in contemporary practice, particularly for advanced hybrid and branched endovascular techniques. High-volume centers with dedicated aortic programs are more likely to achieve favorable outcomes across all modalities.

**Figure 3:**
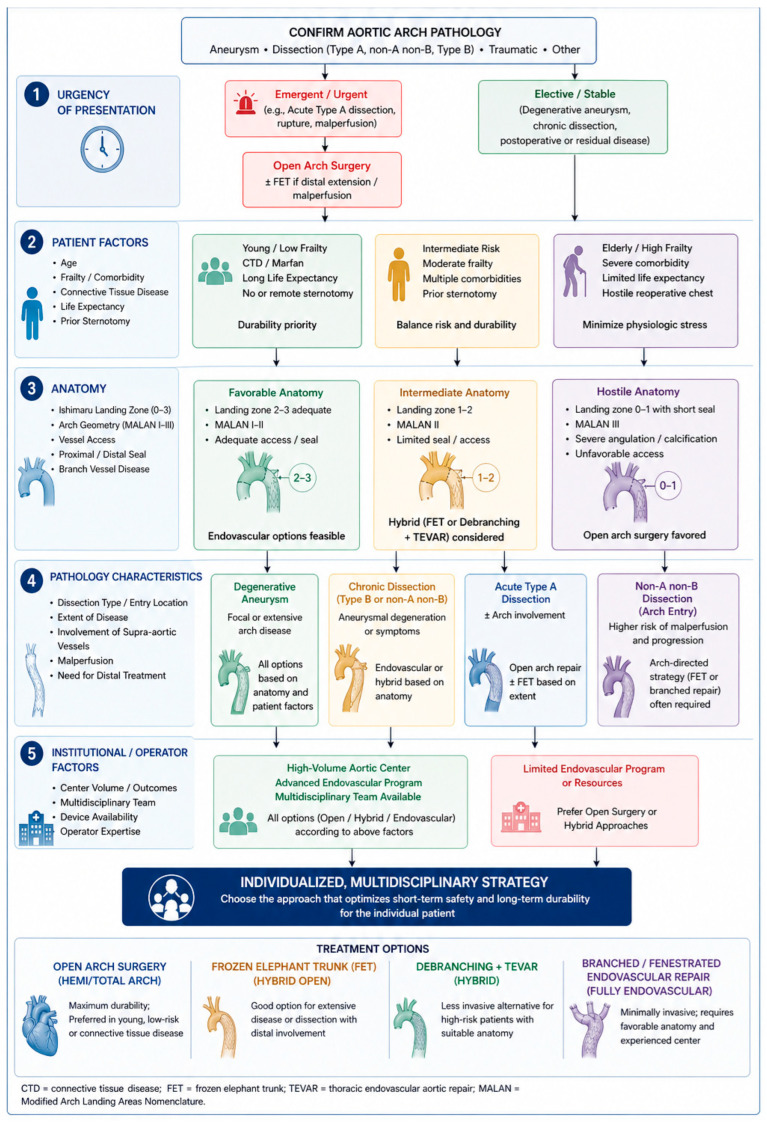
Decision-making framework for management of the aortic arch.

Taken together, these variables support a tailored, multidisciplinary approach in which open, hybrid, and fully endovascular strategies are selected not in isolation but as part of an integrated treatment pathway aligned with patient risk, anatomic complexity, and long-term goals [1,27]. A broad decision-making framework is presented in Figure 3.

**Table 1:** Contemporary Management Options for the Aortic Arch.

Management Option	Typical Indications	Anatomic Conditions	Neurologic Risk	Durability and Reintervention	Notes
**Open hemi/** **Total arch** **replacement (OAR)**	Young/fit patients; connective-tissue disease; hostile arch geometry; need for extensive proximal/distal reconstruction or root work	Detailed CT planning; arch zone definition; cannulation strategy for ACP; plan distal landing for future TEVAR if staged	Selective antegrade cerebral perfusion with moderate/low hypothermia supported by RCT and contemporary data [3,15]	Benchmark durability; better 3–5 yr survival vs. debranching HAR, at the expense of higher perioperative physiologic stress [23,24]	Not device-specific; tailored to center expertise
**Hybrid—Frozen** **Elephant Trunk (FET)**	Complex arch ± proximal descending pathology; type A/B dissections with distal malperfusion; arch aneurysm requiring distal landing	Surgical arch graft with integrated distal stent (creates landing for later TEVAR); consider zone-2 arch strategies and spinal-cord protection [22]	Stroke/SCI risks mitigated with refined perfusion and shorter coverage; careful planning to avoid distal stent-induced new entry [20,22]	Increasingly favorable early/mid-term outcomes; allows for staged distal endovascular completion	Thoraflex Hybrid, E-vita; large single-center/registry series over 10–15 yrs
**Hybrid—** **Debranching** **+ TEVAR (HAR)**	Older/high-risk patients with arch aneurysm where sternotomy/CPB risk is high; need to preserve supra-aortic flow with extra-anatomic bypass	Plan LSA revascularization when landing in Zone 2; assess arch curvature/landing zones; staged vs. single-stage strategy [28]	Procedural stroke risk present (manipulation + arch coverage); embolic protection strategy varies by center	Similar short-term survival to OAR but higher late reintervention [23,24]	Extra-anatomic bypass (e.g., carotid–subclavian, carotid–carotid) + standard TEVAR platforms
**Fully** **endovascular—** **single-branch arch devices**	Degenerative arch lesions or select dissections in pts unfit for open/hybrid; centers with dedicated arch endovascular programs	Strict anatomical criteria (ascending diameter/length, arch angulation, branch takeoff, seal zones); routine LSA revascularization when Zone 2 covered	30-day stroke rates generally moderate-to-low in experienced centers [29,30]	Mid-term durability evolving; requires surveillance for branch patency/endoleak [29]	Gore TAG TBE—U.S. FDA: Zone 2 (earlier) and expanded to Zones 0–1 in 2025 [5]
**Fully** **endovascular—** **double-branch or** **modular arch** **systems**	Extensive arch involvement requiring both innominate + LCCA perfusion via branches; centers with trial access	Requires supra-aortic debranching in some configurations; meticulous measurements; often under clinical trial/registry	Early series/registries show high technical success with acceptable early stroke/mortality [25,26]	Durability still emerging; reintervention/endoleak monitored in registries [25]	Nexus/Nexus Duo (Endospan)—2025 Italian registry: ~97% technical success, ~6–7% peri-op mortality/stroke. RelayBranch (Terumo)—U.S. early-feasibility/Breakthrough Device; multicenter trials ongoing [4]

**ACP**: Antegrade Cerebral Perfusion; **RCT**: Randomized Controlled Trial; **OAR**: Open Arch Replacement; **HAR**: Hybrid Arch Repair; **FET**: Frozen Elephant Trunk; **SCI**: Spinal Cord Ischemia; **TEVAR**: Thoracic Endovascular Aortic Repair; **CPB**: Cardiopulmonary Bypass; **LSA**: Left Subclavian Artery; **LCCA**: Left Common Carotid Artery. The use of bold was made to highlight the options.

## 7. Outcomes

Interpretation of outcomes in aortic arch management must be contextualized by the heterogeneity of the available evidence. While open surgical repair is supported by relatively mature datasets, including large observational series and comparative analyses, evidence for hybrid and fully endovascular approaches is more limited, often derived from single-center experiences, registries, and early feasibility studies with shorter follow-up. As such, comparisons across modalities should be interpreted with caution.

### 7.1. Open Arch Replacement

While surgical repair remains the first-line therapy for patients with aortic disease involving the arch, the rate of perioperative mortality and neurological complications differs in elective and emergency situations. A recent single-center study reported 30-day mortality of 7.6% and major neurologic complications of 6.8% overall [31]. Outcomes are influenced by the extent of repair, with higher perioperative risk observed in total arch replacement compared with hemiarch procedures, although both remain within acceptable benchmarks.

Longer-term data further support the durability of open repair. Comparative analyses suggest improved freedom from late aortic reintervention following total arch replacement compared with hybrid strategies up to six years postoperatively, particularly in younger patients and those with connective tissue disease [23]. These findings are supported by relatively mature follow-up data and reinforce the role of open repair as the current benchmark against which other approaches are measured.

The incorporation of the FET technique within OAR has further expanded its applicability. Early and mid-term outcomes from multicenter and single-center studies are encouraging, with acceptable mortality and neurologic complication rates. The U.S. multicenter Thoraflex trial demonstrated positive 1-year results with 5% permanent stroke and paraplegia rates, and a 15-year experience with hybrid grafts showed a sustained survival rate of 66% with device-specific nuances [32]. However, long-term durability beyond intermediate follow-up remains less well defined, particularly in comparison to conventional open repair without distal stent grafting.

### 7.2. Fully Endovascular Arch Repair (Branched/Fenestrated)

Fully endovascular approaches to the aortic arch have progressed from conceptual feasibility to early clinical application in selected patients and specialized centers. Nana et al. reported 94.4% technical success, 11.9% 30-day mortality, and 13.5% stroke for a single-center cohort of 126 patients treated with customized fenestrated/branched devices in zones 0–2, and 46.4% independence from reintervention at 24 months [25]. Another study found technical success, perioperative mortality, and stroke rates were similar in both branched and fenestrated devices [26].

However, the current evidence base remains limited and should be interpreted cautiously. Most available data are derived from small, highly selected cohorts, single-center experiences, and registry-based analyses, often with relatively short follow-up durations. Neurologic complications, particularly stroke, remain a significant concern, especially with more proximal arch interventions. Additionally, rates of secondary reintervention remain substantial, reflecting ongoing challenges related to device durability, endoleak, and branch vessel patency [25,26].

According to a comprehensive analysis of the Gore TAG TBE, stroke was detected in 2.5% of cases at 30 days and in 5.9% of cases at 12 months following zone-2 landing. Despite continuous post-approval monitoring, preliminary data for enlarged zone 0–1 indication demonstrate satisfactory 30-day safety in crucial cohorts. Another study from Canada with 11 patients (zones 0–2) reported no branch instability, spinal cord paralysis, stroke in elective cases, or deaths after 7 months of imaging [5,30,33]. Nevertheless, these technologies remain dependent on careful patient selection and advanced operator expertise, and their long-term durability relative to open repair has yet to be established.

Overall, while fully endovascular strategies are rapidly expanding the therapeutic landscape of aortic arch disease, they remain best viewed as evolving technologies, with outcomes that are highly center-dependent and supported primarily by early- to mid-term data.

## 8. Limitations

This review has several limitations. First, it is a narrative review and does not follow a formal systematic methodology; therefore, selection of included studies may be subject to bias and may not fully capture all available evidence. Second, the literature on contemporary aortic arch management is heterogeneous, comprising a mix of randomized data, observational studies, single-center experiences, and registry-based analyses, which limits direct comparison across treatment strategies. Third, many of the data points informing hybrid and fully endovascular approaches are derived from early feasibility studies and high-volume centers, which may affect generalizability. Finally, the rapid evolution of endovascular technologies and techniques means that current evidence may not fully reflect future practice. These considerations should be taken into account when interpreting the findings of this review.

## 9. Conclusions and Future Directions

Management of the aortic arch is increasingly becoming patient-specific and zone-driven with different options including open, hybrid and fully endovascular strategies. Open hemi- or total arch replacement remains the standard for durability, especially in younger patients and those with heritable thoracic aortic disease. Hybrid approaches, particularly the FET, play a central role by bridging open and endovascular paradigms, especially in patients with combined arch and descending pathology or dissection with distal involvement. Fully endovascular arch repair is emerging as a viable option in carefully selected patients with favorable anatomy, particularly in higher-risk populations treated at experienced centers, although long-term durability remains less well established.

Across all strategies, outcomes depend on thorough preprocedural planning and structured postoperative surveillance. Contemporary aortic arch management is therefore best approached as an individualized, multidisciplinary process aligned with patient risk, anatomic complexity, and long-term goals.

## Data Availability

No new data were created or analyzed in this study.

## Abbreviations

The following abbreviations are used in this manuscript:

Frozen elephant trunk	FET
Thoracic endovascular aortic repair	TEVAR
Modified Arch Landing Areas Nomenclature	MALAN
American College of Cardiology/American Heart Association	ACC/AHA
European Society of Cardiology	ESC
Left subclavian artery	LSA
Type, Entry, Malperfusion	TEM
European Association for Cardio-Thoracic Surgery/Society of Thoracic Surgeons	EACTS/STS
Society for Vascular Surgery/Society of Thoracic Surgeons	SVS/STS
Hypothermic circulatory arrest	HCA
Hybrid arch repair	HAR
Native proximal aortic landing	NPAL
Penetrating aortic ulcer	PAU
Intramural hematoma	IMH
Antegrade Cerebral Perfusion	ACP
Randomized Controlled Trial	RCT
Open Arch Replacement	OAR
Spinal Cord Ischemia	SCI
Cardiopulmonary Bypass	CPB
Left Common Carotid Artery	LCCA
